# Supplementary material for: Effect of Impulsivity Traits on Food Choice within a Nudging Intervention
Source: Nutrients. 2020 May 14;12(5):1402. doi: 10.3390/nu12051402 (PMC7285079; doi:10.3390/nu12051402)
Supplement: Supplementary file 1 [file nutrients-12-01402-s001.pdf]

Supplementary Material - Appendix A

Complete questionnaire:

Welcome to our study

Thank you for participating! Please follow the instructions below:

STEP 1: Fill out the attached consent form and keep a copy for yourself.

STEP 2: Fill out section #1, #2, and #3.

STEP 3: Eat! Please remember to check in your bracelet every time before serving yourself at each different food section. You are welcome to eat as much as you like.

STEP 4: After finishing your meal, please fill out section #4 and hand it in to one of us.

Section #1: BEFORE eating

1. What is your gender?
   - ❑ Female
   - ❑ Male

2. How old are you?

   ____

3. What is your occupation?
   - ❑ Student
   - ❑ Employed
   - ❑ Unemployed

4. What is your highest level of education?
   - ❑ High school or lower
   - ❑ Bachelor
   - ❑ Master degree

- ❑ PhD or higher

5. How tall are you (in cm)?

____

6. How much do you weigh (in kg)? If you do not know the exact answer, you just write an estimate.

____

7. How would you classify yourself in terms of your food consumption?
   - ❑ Omnivore
   - ❑ Flexitarian
   - ❑ Vegetarian
   - ❑ Pescetarian
   - ❑ Vegan
   - ❑ I do not know

8. Have you changed any of the following habits in the last two months: diet, smoking or exercising?
   - ❑ Yes
   - ❑ No

Section #2 - BEFORE eating

Below are a number of statements that describe ways in which people act and think. For each statement, please indicate how much you agree or disagree with the statement. If you **Agree Strongly** circle **1**, if you **Agree Somewhat** circle **2**, if you **Disagree Somewhat** circle **3**, and if you **Disagree Strongly** circle **4**. Be sure to indicate your agreement or disagreement for every statement below.

	**Agree Strongly**	**Agree Somewhat**	**Disagree Somewhat**	**Disagree Strongly**
1. I generally like to see things through to the end.	**1**	**2**	**3**	**4**
2. My thinking is usually careful and purposeful.	**1**	**2**	**3**	**4**
3. When I am in a great mood, I tend to get into situations that could cause me problems.	**1**	**2**	**3**	**4**
4. Unfinished tasks really bother me.	**1**	**2**	**3**	**4**
5. I like to stop and think things over before I do them.	**1**	**2**	**3**	**4**
6. When I feel bad, I will often do things I later regret in order to make myself feel better now.	**1**	**2**	**3**	**4**
7. Once I get going on something I hate to stop.	**1**	**2**	**3**	**4**
8. Sometimes when I feel bad, I can’t seem to stop what I am doing even though it is making me feel worse.	**1**	**2**	**3**	**4**
9. I quite enjoy taking risks.	**1**	**2**	**3**	**4**
10. I tend to lose control when I am in a great mood.	**1**	**2**	**3**	**4**
11. I finish what I start.	**1**	**2**	**3**	**4**
12. I tend to value and follow a rational, "sensible" approach to things.	**1**	**2**	**3**	**4**
13. When I am upset I often act without thinking	**1**	**2**	**3**	**4**
14. I welcome new and exciting experiences and sensations, even if they are a little frightening and unconventional.	**1**	**2**	**3**	**4**
15. When I feel rejected, I will often say things that I later regret.	**1**	**2**	**3**	**4**
16. I would like to learn to fly an airplane	**1**	**2**	**3**	**4**
17. Others are shocked or worried about the things I do when I am feeling very excited.	**1**	**2**	**3**	**4**

18. I would enjoy the sensation of skiing very fast down a high mountain slope.	**1**	**2**	**3**	**4**
19. I usually think carefully before doing anything	**1**	**2**	**3**	**4**
20. I tend to act without thinking when I am really excited	**1**	**2**	**3**	**4**

Section #3: BEFORE eating

Please answer these questions BEFORE you start to eat.

9. **How hungry do you feel now?** Please circle a number on the scale below.

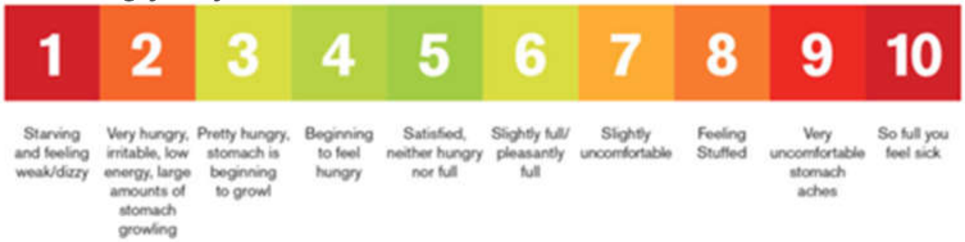

10. **How much do you think you can eat?** Please indicate how much you can eat on a scale of 1 to 10, from **nothing at all = 1** to **a lot = 10**. Please circle a number on the scale below.

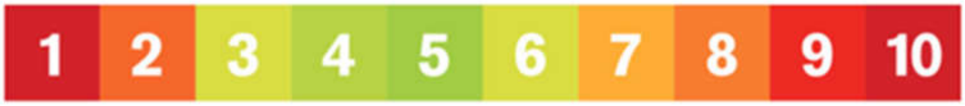

Section #4: AFTER eating

Please answer these questions AFTER you have finished eating.

11. **How satiated are you?** Please circle a number on the scale below.

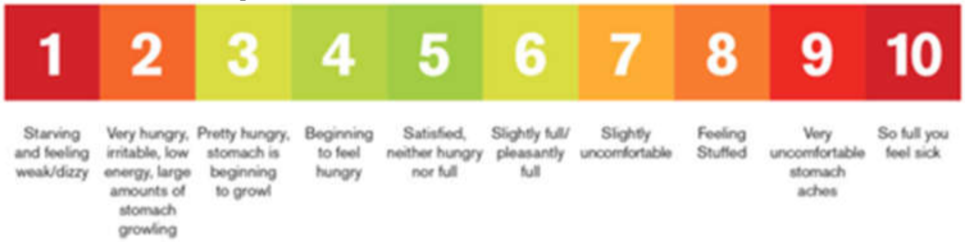

12. **How much did you like the food available at the buffet?** Please indicate how much you liked the food on a scale of 1 to 10, from **I didn't like it at all = 1** to **I liked it a lot = 10.** Please circle a number on the scale below.

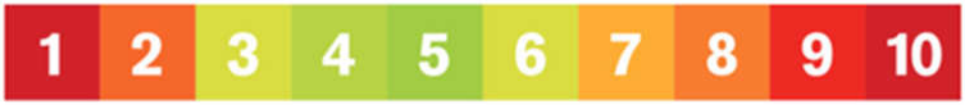

Thank you for your time.

Have a nice day!

Supplementary Material - Appendix B

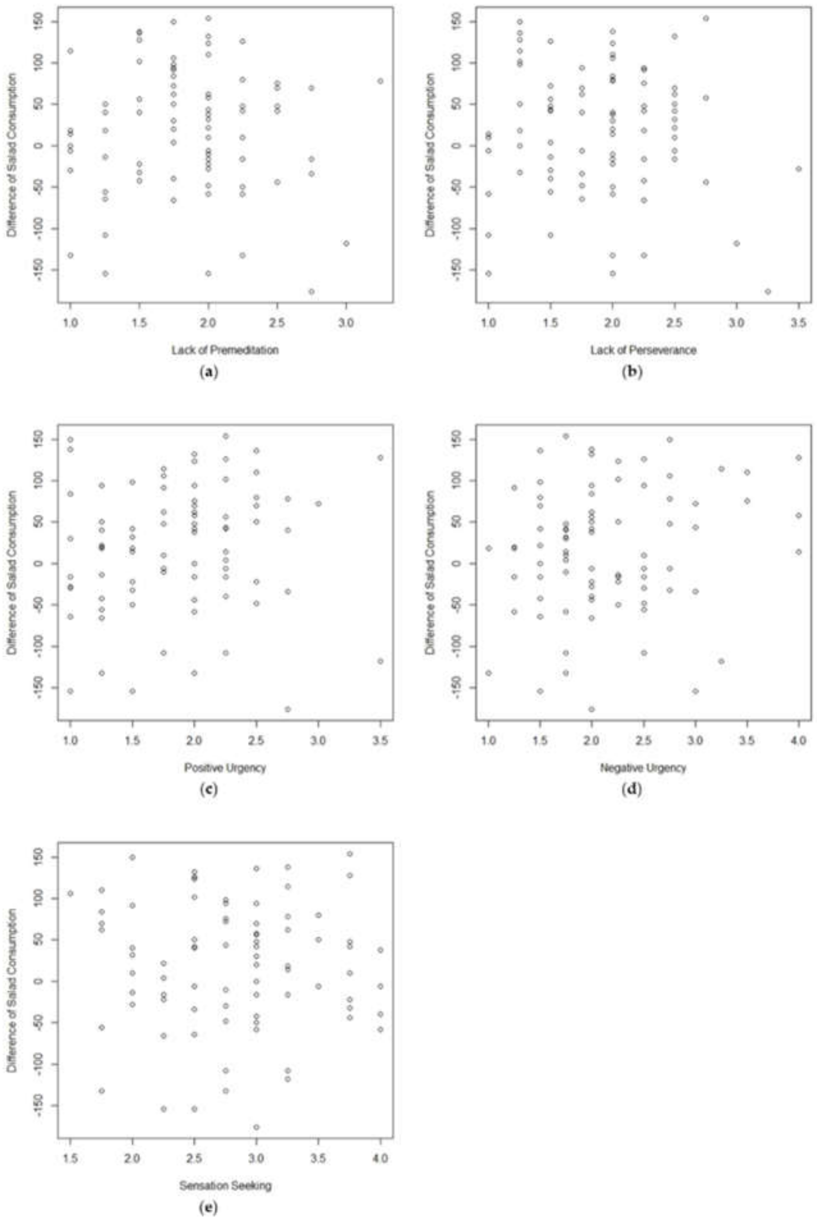

Figure B1. Scatter plots of each impulsivity trait score VS the difference of salad consumption (g) between lunch settings; (a) Lack of Premeditation; (b) Lack of Perseverance; (c) Positive Urgency; (d) Negative Urgency; (e) Sensation Seeking.

Table B1. Energy content for each ingredient of the meal components. Retrieved from Matportalen and from some of the product´s nutrition labels

Ingredients	Energy (kcal)/100g
Pasta	150
Olive oil	889
Cucumber	10
Tomato	18

Zucchini	17
Broccoli	30
Cauliflower	23
White cabbage	32
Lettuce	16
Ground meat	200
Mushrooms	22
Eggplant	20
Carrot	36
*Tomato sauce	27
Sugar	400
*Bouillon	293

*Energy content taken from the product´s nutrition label

Table B2. Recipe for tomato sauce

	g	%
Canned tomato sauce	3750	75
Sugar	40	0.8
Bouillon	10	0.2
Water	1200	24
Total	**5000**	**100**

Table B3. Calculations of energy content of meal components

	Ingredients	% in formula	Quantity	Kcal	
Pasta	pasta	99	99	148.5	
	oil	1	1	8.89	
	**Total**			**157.39**	**kcal for each 100g of pasta**
	cucumber	0.16	16	0.80	
	tomato	0.16	16	1.45	
	zucchini	0.15	15	1.28	
Salad	broccoli	0.12	12	1.81	

	cauliflower	0.12	12	1.39	
	white cabbage	0.2	20	3.22	
	lettuce	0.09	9	0.72	
	**Total**	1	100	**10.67**	**kcal for each 100g of salad**
**Meat sauce**					
	meat	0.3	30	60	
vegetables	0.2		20		
	broccoli	20	4	1.2	
	mushrooms	25	5	1.1	
	eggplant	25	5	1	
	carrot	30	6	2.16	
				**5.46**	
sauce	0.5		50		
	tomato sauce	75.00	37.50	10.13	
	sugar	0.80	0.40	1.60	
	bouillon	0.20	0.10	0.29	
	water	24.00	12.00	0.00	
				**12.02**	
	**Total meat sauce**			**77.48**	**for each 100 g of meat sauce**
**Vegetable sauce**					
	0.5		50		
	broccoli	20	10	3	
	mushrooms	25	12.5	2.75	
vegetables	eggplant	25	12.5	2.5	
	carrot	30	15	5.4	
				**13.65**	
	0.5		50		
	tomato sauce	75.00	37.50	10.13	

sauce	sugar	0.80	0.40	1.60	
	bouillon	0.20	0.10	0.29	
	water	24.00	12.00	0.00	
				**12.02**	
	**Total vegetable sauce**			**25.67**	**for each 100 g of vegetable sauce**
